# Ultrasound-guided microwave ablation in the treatment of early-stage tongue cancer

**DOI:** 10.3389/fonc.2022.950228

**Published:** 2022-08-30

**Authors:** Jianquan Yang, Wen Guo, Rong Huang, Zhengmin Xu, Chunyang Zhou, Man Lu

**Affiliations:** ^1^ The School of Medicine, University of Electronic Science and Technology of China, Chengdu, China; ^2^ Department of Ultrasound Medical Center, Sichuan Cancer Hospital and Institute, Sichuan Cancer Center, School of Medicine, University of Electronic Science and Technology of China, Chengdu, China; ^3^ Institute of Materia Medica, North Sichuan Medical College, Nanchong, China

**Keywords:** ultrasound, microwave ablation, early-stage, tongue cancer, treatment

## Abstract

**Background:**

Tongue cancer is a common malignant tumor of the head and neck. Its treatment methods include surgery, radiotherapy, and chemotherapy. However, these treatments have serious side effects and poor cosmetic effect, so it is urgent to find new treatment methods. We pioneered the use of microwave ablation (MWA) in the treatment of early tongue cancer and achieved good results.

**Case Presentation:**

A 67-year-old woman (Han nationality) was admitted to the hospital because of progressive aggravation of tongue pain. She had a history of tongue pain of more than 1 year. Pathological biopsy showed squamous cell carcinoma; following this, radical operation of the tongue cancer was planned. The preoperative examination showed thyroid occupation in the upper mediastinum region compressing the airway; hence, the risk of general anesthesia was high. Consent was obtained from the patient and her family. Ultrasound-guided MWA was successfully performed under the lingual nerve block. The patient was followed for 1 year. She recovered well with no dysphagia and unclear articulation symptoms, and the cosmetic effect was excellent.

**Conclusion:**

To our knowledge, this is the first case of using MWA for the treatment of early-stage tongue cancer (ESTC). Ultrasound-guided MWA may be used for ESTC that can completely ablate the tumor and retain the function of the tongue, further improving the quality of life of the patient. However, it is only a case report and needs more research to verify the use of MWA in ESTC.

## Introduction

Oral cancer is considered one of the most common malignant tumors of the head and neck. Recent statistical data on oral cancer have shown it to be increasing year by year and accounting for about 4% (1); tongue cancer accounts for a large proportion of oral cancers. Surgery is considered one of the major treatments for tongue cancer, while patients with advanced stage need postoperative radiotherapy and chemotherapy. The major drawback of radical resection of tongue cancer is trauma, which in turn affects the patient’s daily life. This adversely affects vital functions of patients such as swallowing, chewing, and speaking. Treatments such as postoperative radiotherapy and chemotherapy will further aggravate these side effects. The 5-year survival rate of these patients is found to be 50%–60% (2), while there is no significant improvement of the survival rate in the past 10 years (3). It is urgent to find new treatments to improve the prognosis of patients with tongue cancers.

With the progress of modern imaging technology, microwave ablation (MWA) has been applied in the treatment of a variety of solid tumors, such as liver cancer, thyroid papillary carcinoma. MWA has been proven to be safe, effective, and minimally invasive (4–6). However, the use of MWA in the treatment of tongue cancer, especially in early-stage tongue cancer (ESTC), has not been reported elsewhere. Herein, we report a case of ultrasound-guided MWA in the treatment of ESTC.

## Case presentation

A 67-year-old woman (Han nationality) was admitted to the hospital because of progressive aggravation of tongue pain. She had a history of tongue pain of more than 1 year. Pathological biopsy showed squamous cell carcinoma; following this, radical operation of the tongue cancer was planned. The preoperative examination showed thyroid occupation in the upper mediastinum region compressing the airway; hence, the risk of general anesthesia was high. The doctor convinced them of the issue and advised them to undergo MWA; as a result, consent was given by the patient and family members.

### Preoperative examination

**Figure 1:**
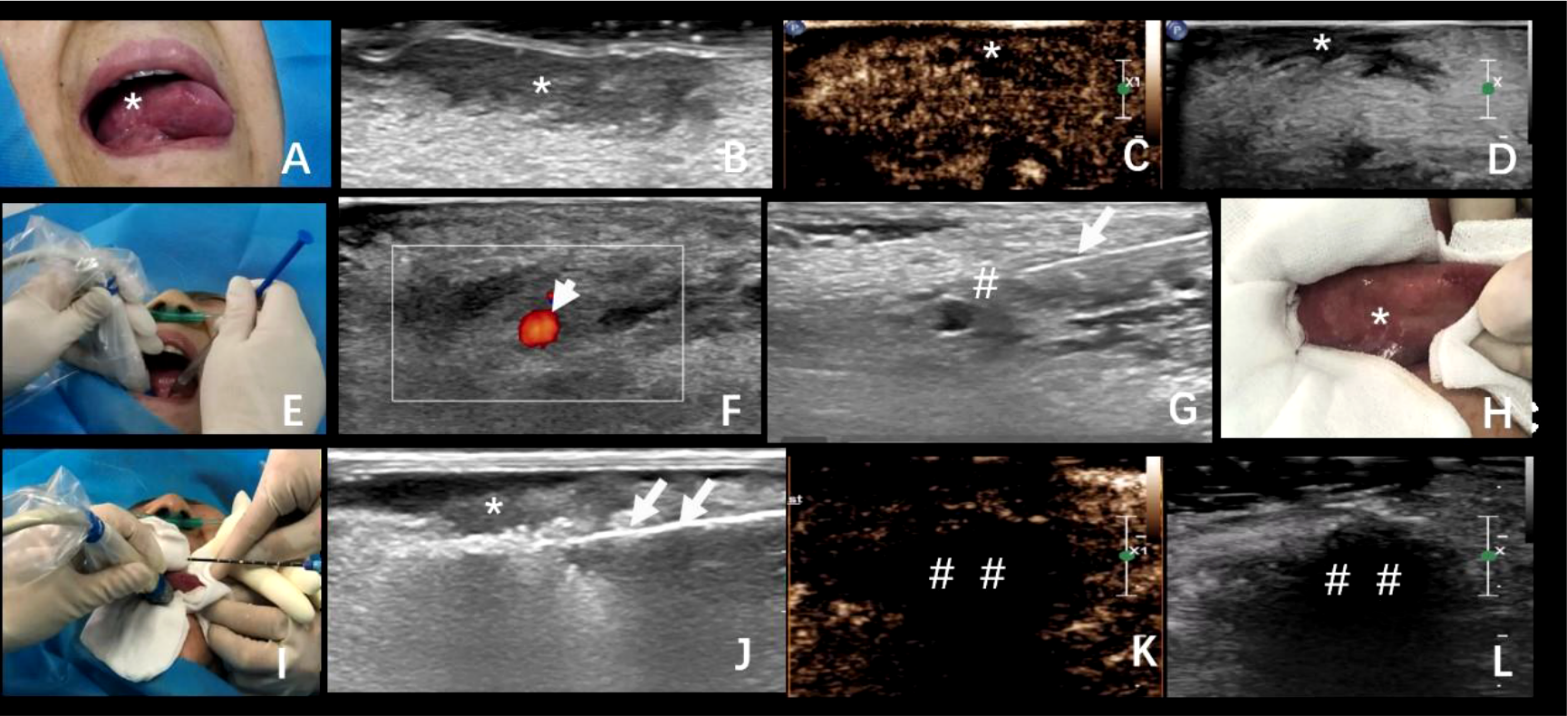
**(A)** A 67-year-old woman with progressive aggravation of tongue pain, and her lesion was confirmed in pathology as squamous cell carcinoma. **(A, B)** Tumor location and ultrasonic image. **(C, D)** Preoperative contrast-enhanced ultrasonography of the tumor. **(E–G)** The process of lingual nerve block guided by ultrasound. **(H)** Protection of the surrounding mucosa before ablation. **(I, J)** The process of ultrasound-guided microwave ablation. The double arrowhead: the ablation needle. **(K, L)** The area of ablation showed no enhancement in the arterial phase and venous phase after ablation. *The exact location of the tumor; ^#^lingual nerve block area; long arrow: puncture needle; ^##^The area of ablation. Short arrow: Lingual artery; Long arrow: Puncture needle; The double arrowhead: The ablation needle.

There was no abnormality in the blood routine, liver and kidney function, blood coagulation, and ECG. The Ultrasound was performed using Philips EPIQ 7 ultrasound system (10 MHz endocavitary transducer, Bothell, WA, USA) to discover the belly of the right tongue. The tongue was rough, and ulcerative changes of about 0.6 cm in diameter were also observed at about 1 cm from the root of the tongue; there was no obvious tenderness or bleeding by touch (**Figure 1A**).

### Ablation process

The consent was obtained from the patient and her family; the ablation was performed on 20 April 2020. The operation procedure was performed by the doctor with 10 years of experience in interventional ultrasound. First of all, the puncture path was confirmed by traditional and contrast-enhanced ultrasound; this showed low enhancement in the arterial phase and venous phase of the lesion (**Figures 1B–D**). Further with appropriate sterilization, the patient was given local anesthesia *via* the lingual nerve block with a mixture of 2% lidocaine and ropivacaine (2 ml) (**Figures 1E–G**). Moreover, during the procedure, critical care has been taken to protect the oral mucosa and normal tongue tissue by ice physiological saline bag (normal saline, 50 ml) with soaked sterile gauze (**Figure 1H**). Then, the microwave antenna (22G) (model: KY-2450A-1, Kangyou Medical, Nanjing, China) was advanced to the predetermined position in the target hypoechoic nodule by ultrasound guidance. The ablation needle was inserted into the tongue, and the ablation was expanded from the base of the low echo mass to the shallow surface. The output power was 30 W during the procedure (**Figures 1I, J** **)**, and the operation time lasted for about 10 min. Contrast-enhanced ultrasound showed no enhancement in the arterial phase and venous phase after ablation by injection of 2.4 ml sulfur hexafluoride (**Figures 1K, L** **)**. After ablation, the gasification range exceeded the boundary of the mass by 1.5 cm. The score of numeric rating scale (NRS) intraoperatively was 0, and 8 h after ablation, the score was 3. Soon afterward, the patient reported to be relieved of pain after ice compress, and there was no symptom of fever or difficulty in chewing.

### Postoperative follow-up

**Figure 2:**
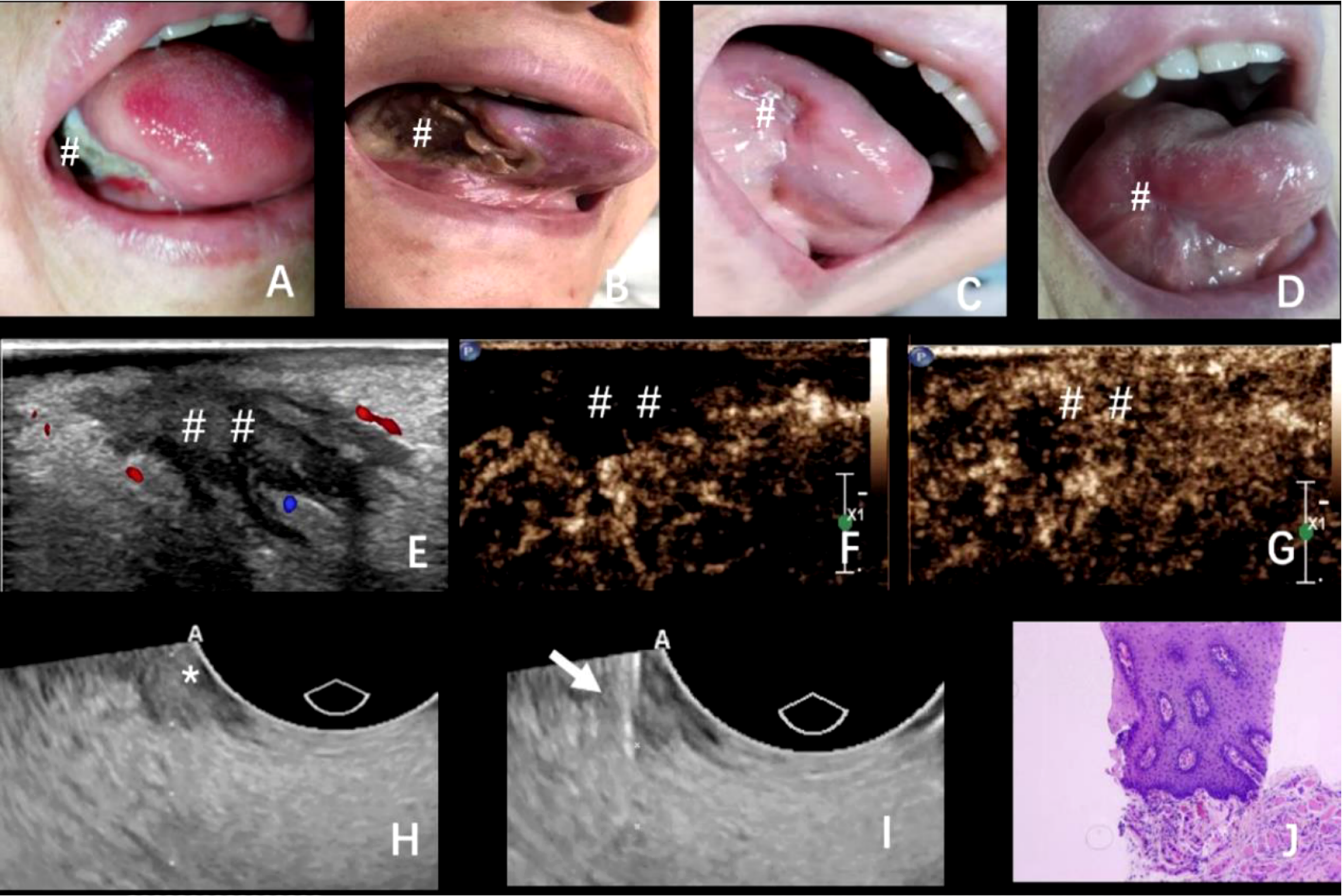
**(A–D)** The changes in the ablation area after the operation at 4, 7, and 21 days and 2 months; **(E–G)** 2 months after the operation, ultrasound examination showed that the ablation area showed patchy low echo, clear boundary, irregular shape, and punctate blood flow signal. Contrast-enhanced ultrasound showed that the ablation area showed slow and low enhancement in the arterial phase and equal enhancement in the venous phase; **(H, I)** Puncture biopsy of different parts of hypoechoic area under the guidance of intracavitary ultrasound; **(J)** Pathological biopsy showed that no cancer cells were found in the ablation area. The arrow: Puncture biopsy needle; * and ^#^The exact location of the lesions; ^##^The area of ablation.

**Figure 3:**
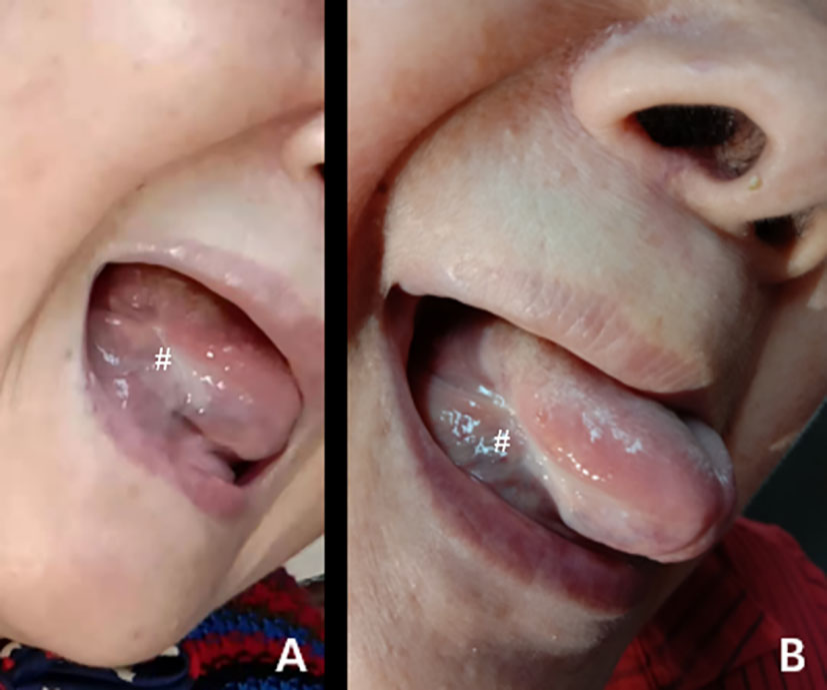
**(A, B)** The changes in the ablation area after the operation at 6 months **(A)** and 1 year **(B)**. ^#^The exact location of the lesions.

Four days after ablation, a scab was formed in the ablation area that was light brown in color (**Figure 2A**). One week later, the crust fell off with bleeding and hemostasis outside the hospital (**Figure 2B**). Three weeks later, the crust completely peeled off with mucous membrane growth (**Figure 2C**). Two months later, scars were formed in the ablation area, with no restriction of tongue movement and difficulty in chewing (**Figure 2D**). Further follow-up using contrast-enhanced ultrasound in the ablation area revealed slow and low enhancement in the arterial phase and equal enhancement in the venous phase (**Figures 2E–G**). In order to verify the complete ablation of the tumor, we used intraluminal ultrasound-guided puncture biopsy in different parts of the hypoechoic area (**Figures 2H, I** **)**. Moreover, the pathological biopsy showed no cancer cells in the ablation area (**Figure 2J**). Six months (**Figure 3A**) and 1 year later (**Figure 3B**), the patient recovered well after ablation, with no restriction of tongue movement and chewing.

## Discussion

Tongue cancer as a common oral malignant tumor, due to the lack of specific clinical symptoms in the early stage, is difficult to distinguish from oral benign diseases. Most of them are diagnosed in the advanced or late stage. It is also noted that the 5-year survival rate of these patients is always less than 60%. Therefore, it is urge to find a new treatment strategy to increase the efficiency in early diagnosis and improve survival time.

CT and MRI are common examination methods for oral diseases, especially tumors, but some early-stage cases and some oral metal foreign bodies cannot be diagnosed qualitatively (7). As a routine imaging examination, ultrasound imaging plays an important role in the staging and restaging of head and neck tumors (8, 9). However, ultrasound images are not much efficient to diagnose the deep position of the oropharynx and floor of the mouth by body surface scanning. In our previous research, the intraluminal probe (frequency 10 MHz) was used to display the tumor location, and puncture biopsy was carried out under the guidance of ultrasound, which improved the positive rate of tumor diagnosis (10). Hence, in this case, we had used intracavitary ultrasound probe, which can clearly show the tumor location and boundary, providing images for tumor ablation.

Surgery is considered the main treatment in tongue cancer patients, while patients with advanced stage undergo postoperative radiotherapy and chemotherapy (11, 12). Radical resection of tongue cancer causes great trauma to the patients and changes the normal anatomical structure and reduces the quality of life of the patients. Moreover, postoperative radiotherapy will further aggravate the patients’ speaking and chewing. As a minimally invasive treatment, MWA has the same survival time compared with surgery in hepatocellular carcinoma, especially with diameters less than 3 cm. MWA was also performed in thyroid papillary carcinoma, and the result showed a better therapeutic and cosmetic effect (13–15). In this patient, because of her thyroid occupation, general anesthesia could not be performed. In lingual soft tissue anesthesia, the most commonly used technique is inferior alveolar nerve block, which can induce potential complications such as nerve injury, occlusion, facial nerve paralysis. Some researchers have proven that lingual nerve block is better than inferior alveolar block in oral disease, which can reduce the incidence of abnormal sensation of the lingual nerve and difficulty in mouth opening (16). Therefore, in this case, we used lingual nerve block to anesthetize. After anesthesia, the ablation process was performed successfully and no obvious pain in the process of ablation.

Radical resection of the tongue cancer is the main treatment of tongue cancer, but after the operation, it will seriously affect the patients’ functions of speaking and chewing and significantly decrease the quality of life of the patients. At the same time, postoperative radiotherapy will further aggravate the patients’ speaking and swallowing function (17–19). As a minimally invasive treatment, MWA not only kills the tumor but also retains the integrity of organs and improve the cosmetic effect. In this case, moderate pain occurred within 8 h after the operation and relieved after ice compress. During the postoperative follow-up, we found that scab was formed gradually in the ablation area. One month later, a scar was formed in the ablation area, and there were no symptoms of dysphagia and unclear articulation. In early-stage liver cancer, MWA is carried out for those patients who cannot tolerate surgery. Studies have also shown that there is no difference in overall survival time in patients who have undergone MWA or radical surgery (20). However, patients undergoing ablation have a higher local recurrence rate (21), which may be related to the enhancement of invasiveness of residual tumor cells caused by incomplete ablation (22). Two months later, complete ablation was confirmed by puncture biopsy, and the curative effect was evaluated as complete remission (CR). Hence, this case implied that MWA may be safe and effective in the treatment of ESTC.

To our knowledge, this is the first case to explore the use of MWA in the treatment of ESTC. There are a few shortcomings: first, it is a case report; second, the patient’s follow-up time was short. In order to use MWA in the treatment of tongue cancer, multicenter large-sample studies are needed in order prove its efficacy and safety.

## Conclusion

Ultrasound-guided MWA may be used for ESTC, which can completely ablate the tumor and retain the function of the tongue, further improving the quality of life of the patient. However, it is only a case report and needs more research to verify the use of MWA in ESTC.

## Data availability statement

The raw data supporting the conclusions of this article will be made available by the authors, without undue reservation.

## Ethics statement

The studies involving human participants were reviewed and approved by the Ethics Committee of Sichuan Cancer Hospital. The patients/participants provided their written informed consent to participate in this study. Written informed consent was obtained from the individual(s) for the publication of any potentially identifiable images or data included in this article.

## Author contributions

JY, WG and RH carried out the concepts, design, data acquisition, data analysis and manuscript preparation. ZX provided assistance for data acquisition, data analysis. CZ and ML performed manuscript review. All authors contributed to the article and approved the submitted version.

## Funding

This study was supported by funds from National Key Research and Development Program (NO. 2019YFE0196700); Sichuan Medical Association Project (No. S19012).

## Conflict of interest

The authors declare that the research was conducted in the absence of any commercial or financial relationships that could be construed as a potential conflict of interest.

## Publisher’s note

All claims expressed in this article are solely those of the authors and do not necessarily represent those of their affiliated organizations, or those of the publisher, the editors and the reviewers. Any product that may be evaluated in this article, or claim that may be made by its manufacturer, is not guaranteed or endorsed by the publisher.
